# Pin1 promotes pancreatic cancer progression and metastasis by activation of NF‐κB‐IL‐18 feedback loop

**DOI:** 10.1111/cpr.12816

**Published:** 2020-04-29

**Authors:** Qiqing Sun, Guixiong Fan, Qifeng Zhuo, Weixing Dai, Zeng Ye, Shunrong Ji, Wenyan Xu, Wensheng Liu, Qiangsheng Hu, Zheng Zhang, Mengqi Liu, Xianjun Yu, Xiaowu Xu, Yi Qin

**Affiliations:** ^1^ Department of Pancreatic Surgery Fudan University Shanghai Cancer Center Shanghai China; ^2^ Department of Oncology Shanghai Medical College Fudan University Shanghai China; ^3^ Pancreatic Cancer Institute Fudan University Shanghai China; ^4^ Shanghai Pancreatic Cancer Institute Shanghai China

**Keywords:** gene expression, interleukin 18, pancreatic cancer, PIN1

## Abstract

**Objectives:**

Accumulated evidence suggests that Pin1 contributes to oncogenesis of diverse cancers. However, the underlying mechanism of oncogenic function of Pin1 in PDAC requires further exploration**.**

**Materials and Methods:**

IHC was performed using PDAC tissues. Western blot, PCR, immunofluorescence and transwell were performed using cell lines. GSEA were applied for possible downstream pathways. ChIP assay and dual luciferase were used for assessment of transcriptional activity.

**Results:**

Both Pin1 and IL‐18 levels are increased in primary PDAC tissues and that their levels are positively correlated. High expression of IL‐18 is a predictor of poor prognoses. Pin1 promoted pancreatic cancer cell proliferation and motility by increasing IL‐18 expression, while Pin1 knockdown also inhibited the tumour‐promoting effect of IL‐18. Both Pin1 and IL‐18 could enhance the NFκB activity in pancreatic cancer cells. When bound to the p65 protein, Pin1 promoted p65 phosphorylation and its nuclear translocation. In the nucleus, Pin1 and p65 simultaneously bound to the IL‐18 promoter and enhanced IL‐18 transcription. In addition, recruitment of p65 to the IL‐18 promoter was decreased in Pin1‐silenced cells.

**Conclusions:**

Our study improves the understanding of Pin1 in tumour‐promoting inflammation in PDAC, which is a hallmark of cancer; Pin1 interacted with p65 in PDAC and enhanced NF‐κB signalling and downstream transcriptional activation of IL‐18, with increased IL‐18 continuously activating NF‐κB signalling, which then forms a positive feedback loop.

## 1 INTRODUCTION

As one of the most aggressive malignancies worldwide, the death rate for patients with pancreatic ductal adenocarcinoma (PDAC) is nearly equal to its incidence rate.^1^ Due to the late diagnosis and low response to chemotherapy, patients with PDAC have poor survival, and the overall 5‐year survival rate remains at approximately 6%.^2^ Hence, understanding the underlying molecular mechanism of PDAC and finding innovative therapies are needed to conquer these devastating diseases.

Tumour‐associated inflammation, a hallmark of cancer, can contribute to tumorigenesis and progression by providing bioactive molecules in the tumour microenvironment (TME), and this inflammation helps incipient neoplasias acquire other hallmark capabilities.^3^ Nuclear factor kappa‐light‐chain‐enhancer of activated B cells (NF‐κB) has been long suspected to be one of the connections between inflammation and cancer.^4^ There are five members included in the mammalian NF‐κB family: p65 (RelA), RelB, c‐Rel, p50/p105 (NF‐κB1) and p52/p100 (NF‐κB2). The classic NF‐κB pathway includes an extracellular ligand (such as TNFα) binding to its receptor, resulting in activated IκB kinase (IKK) complex.^5^ The function of NF‐κB proteins can be changed through their acquisition of post‐translational modifications, such as phosphorylation, ubiquitination and acetylation; phosphorylation mostly appears as an activating modification. Activated NF‐κB dimers then translocate into the nucleus to activate downstream pro‐inflammatory target genes, such as interleukin‐6 (IL‐6), interleukin‐1 (IL‐1) and interleukin‐8 (IL‐8, CXCL8).^6^ These cytokines can in turn activate NF‐κB signalling, which forms a feedback loop. NF‐κB has an important role in the development of PDAC, and approximately 70% of PDAC tissues showed constitutively activated NF‐κB.^7^ The NF‐κB pathway has been found to promote metastasis in PDAC by regulating epithelial‐mesenchymal transition (EMT) and by regulating angiogenesis factors, such as vascular endothelial growth factor (VEGF).^8^, ^9^ Inhibition of NF‐κB signalling by treatment with Minnelide was also found to lead to deregulation of lymphovascular invasion and neural invasion in a KPC murine model.^10^

As a member of the parvulin subfamily of peptidyl‐prolyl *cis/trans* isomerase, Pin 1 can specifically bind and isomerize phosphorylated Ser/Thr‐Pro peptides in certain proteins.^11^, ^12^ Proline‐directed phosphorylation is a post‐translational modification that regulates various signalling pathways, and its dysregulation could contribute to oncogenic progression.^13^ As Pin1 mediates the isomerization of downstream proteins and changes their structures and activities, it has a pivotal role in multiple processes during tumorigenesis and cancer development.^12^ NF‐κB is a target protein of Pin1. Pin1 has been reported to recognize and bind p65 (Thr254 and Ser276) and induce its phosphorylation, which promotes the activation of downstream molecules.^14^, ^15^ Our previous research has reported that Pin1 is associated with poor prognosis of PDAC. Pin1 promoted pancreatic cancer cell proliferation, which was partially due to mitochondrial dysfunction, and Pin1 also maintained a redox balance via synergistic activation of c‐Myc and NRF2 to counteract *Kras*‐induced mitochondrial respiratory injury.^16^ Another recent study reported that both genetic and chemical ablation of Pin1 dramatically inhibited tumorigenesis and metastasis in a mouse model of PDAC.^17^ However, no study has concentrated on Pin1‐NF‐κB‐mediated tumour progression and metastasis in PDAC. Therefore, we examined the underlying oncogenic mechanism of Pin1 on PDAC.

Interleukin‐18 (IL‐18), which is a pleiotropic member of the IL‐1 family, is synthesized as an inactive 24‐kDa precursor. The intracellular IL‐18 precursor is then cleaved into an 18 kDa mature form by caspase 1 before it is secreted.^18^ Initially identified as an interferon‐γ‐inducing factor in innate and adaptive immune responses, IL‐18 has been reported to enhance Th1 immune responses.^19^ However, IL‐18 is overexpressed in tumour tissues and its expression correlates with tumour progression, metastasis and poor prognosis in some cancers, such as PDAC, extranodal natural killer/T‐cell lymphoma and renal cell carcinoma.^20^, ^21^, ^22^ Evidence has suggested that IL‐18 promotes proliferation and metastasis of pancreatic cancer cell lines through the NF‐κB signalling pathway; however, when co‐administered with BAY11‐7082, an NF‐κB inhibitor, IL‐18 improved survival in a murine model of PDAC.^20^

This study is based on our previous research showing that Pin1 has an oncogenic effect on pancreatic tumorigenesis; here, we examined the relationship between Pin1 and inflammatory chemokines and the mechanism underlying their relationship. We discovered that the expression of Pin1 was positively correlated with the expression of the pro‐inflammatory chemokine IL‐18. Pin1 promoted pancreatic cancer cell proliferation and motility by increasing IL‐18 expression, while Pin1 knockdown also inhibited the tumour‐promoting effect of IL‐18. Both Pin1 and IL‐18 could enhance the NFκB activity in pancreatic cancer cells. We further found that Pin1 may regulate IL‐18 transcription via the NF‐κB pathway. Pin1 and p65 simultaneously bound to and activated the IL‐18 promoter, thus increasing the mRNA levels of IL‐18. In addition, recruitment of p65 to the IL‐18 promoter is decreased in Pin1‐silenced cell lines, which leads to decreased transcription of IL‐18. Collectively, our data support the idea that there is a Pin1‐NF‐κB‐IL‐18 feedback loop, highlight Pin1 as an important treatment target and suggest possible innovative therapies.

## 2 MATERIALS AND METHODS

### 2.1 Cell culture

The human pancreatic cancer cell lines MIA PaCa‐2, Capan‐1 and SW1990 were purchased from the American Type Culture Collection (ATCC, USA) and cultured at 37°C with 5% CO_2_ in a humidified incubator. MIA PaCa‐2 cells were cultured in Dulbecco's modified Eagle's medium with 10% foetal bovine serum (FBS) and 2.5% horse serum. Capan‐1 cells were cultured in Iscove's modified Dulbecco's medium supplemented with 20% FBS. SW1990 cells were maintained in L‐15 medium containing 10% FBS.

### 2.2 Reagents and antibodies

All commercial antibodies and chemicals were purchased from the following sources: anti‐HA (51064‐2‐AP), anti‐NF‐κB p65 (10745‐1‐AP), anti‐Lamin B1 (12987‐1‐AP) and anti‐β‐actin (Proteintech, 60008‐1‐lg) antibodies and human TNFα (HZ‐1014) were manufactured by Proteintech; anti‐Pin1 (ab192036), anti‐NF‐κB p65 (ab19870) and anti‐IL‐18 (ab71495) antibodies were purchased from Abcam; anti‐flag antibody (F1804) was purchased from Sigma; anti‐NF‐κB p65 (Thr254) (11010) was purchased from Signalway Antibody Co.; anti‐NF‐κB p65 (Ser276) (AF3387) antibody was obtained from Affinity Biosciences; and human IL‐18 ELISA kit and recombinant human IL‐18 (rhIL‐18) were purchased from R&D Systems.

### 2.3 Plasmids

pLKO.1 TRC cloning vector (Addgene plasmid 10878) was used to generate shRNA constructs against Pin1 and p65. The 21‐bp target sequences in PIN1 were GCCATTTGAAGACGCCTCGTT and AGGAGAAGATCACCCGGACCA. The 21‐bp target sequences in RELA were GGCGAGAGGAGCACAGATACC and GCATCCAGACCAACAACAACC. pLKO.1‐sh‐scramble (Addgene plasmid 1864) was used as a control plasmid. Flag‐tagged Pin1 was cloned into a pCDH‐CMV‐MCS‐EF vector (System Biosciences), which was used to generate the Pin1 stably overexpressing cells. Pin1‐specific small interfering RNA (siRNA) or negative control siRNA was transfected into pancreatic cancer cells Lipofectamine™ 3000 (Invitrogen).

### 2.4 TCGA data acquisition and statistical analysis

The Cancer Genome Atlas (TCGA)‐PAAD contains RNA expression (Level 3) of pancreatic cancer patients collected from RNA‐seq data by expectation‐maximization; the data were downloaded from the Cancer Genomics Brower of the University of California, Santa Cruz (UCSC; https://genome-cancer.ucsc.edu/).

### 2.5 Gene set enrichment analysis (GSEA)

GSEA version 3.0 (Broad Institute, USA) was used to analyse samples.^23^ We selected 1000 permutations, and Affymetrix was used as the chip platform for calculation of the *P* value and false discovery rate (*q*‐value). All basic and advanced fields were set to default values.

### 2.6 **Quantitative real**‑**time PCR (qRT**‑**PCR)**

The expression levels of the designated genes and β‐actin were determined using an ABI 7900HT Real‐Time PCR System (Applied Biosystems, Inc). The ΔΔC_t_ method was used to determine the relative quantification values of target genes. All reactions were run in triplicate, and primer sequences are listed in Table S1.

### 2.7 Western blotting analysis

Cell pellets were washed twice with ice‐cold PBS and lysed in RIPA buffer on ice. The protein concentration was determined using a bicinchoninic acid protein assay kit (Beyotime). Twenty micrograms of protein from each sample were subjected to electrophoresis on an SDS‐polyacrylamide gel and were then transferred to a polyvinylidene difluoride membrane for subsequent blotting with specific antibodies.

### 2.8 Co‐Immunoprecipitation

Capan‐1 and SW1990 cells were lysed and subjected to immunoprecipitation by overnight incubation of the lysate with a Pin1 antibody and protein A/G beads. IgG was used as a control for each group. Afterwards, beads were washed and boiled to obtain the captured proteins. Samples were then analysed by immunoblotting.

### 2.9 Immunofluorescence staining

Cells were fixed with 4% cold paraformaldehyde and were permeabilized with 0.2% Triton X‐100 before staining for immunofluorescence microscopy analysis. The slides were blocked with 5% bovine serum albumin buffer for 1 hour at 37°C and were then incubated with antibodies at 4°C overnight. Then, they were incubated with Alexa Fluor 488 goat anti‐mouse IgG or rhodamine (TRITC) goat anti‐rabbit antibodies for 1 hour at 37°C. DAPI (Sigma) was applied to stain nuclear DNA. A Leica TCS SP5 confocal microscope was used to capture images.

### 2.10 Wound healing assay and transwell migration and invasion assay

Wound healing assays were performed as described previously.^24^ Equal numbers of cells (~6 × 10^4^ cells) were suspended in 200 μL of serum‐free medium before seeding in the upper chamber with or without Matrigel. The 20‐well culture plate was filled with 800 μL of medium supplemented with 10% FBS. After 24 hours of cell culture, the cells in the lower chamber were washed, fixed and stained. The number of cells was calculated in randomly selected fields.

### 2.11 Clinical samples and immunohistochemical (IHC) staining

The clinical tissue samples in this study were obtained from patients who were diagnosed with PDAC at Fudan University Shanghai Cancer Center. The study was approved by the Institutional Research Ethics Committee of Fudan University Shanghai Cancer Center, and informed consent was obtained from each patient. IHC staining for PIN1 and IL‐18 was performed according to standard procedures, as described previously.^25^ The strict pathological diagnoses were made by two independent pathologists. Protein expression was evaluated by multiplying the positivity (0, <5%; 1, 5%‐25%; 2, 25%‐50%; 3, 50%‐75%; 4, >75%) and staining intensity (0, no coloration; 1, pale yellow; 2, yellow; and 3, dark yellow). The expression levels were classified as follows: negative (0‐3, −); weakly positive (4, +); moderately positive (6, ++); and strongly positive (>6, +++).

### 2.12 Promoter activity assessment by dual‐luciferase assay

The luciferase reporter construct pGMNF‐κB‐Lu (GM‐021001, Genomeditech) was transiently cotransfected with a vector‐expressing Pin1 into Capan‐1 and SW1990 cell lines grown on 96‐well plates using Lipofectamine™ 3000 (Invitrogen). The IL‐18 promoter region, spanning from −2500 to 250 of the transcription starting site, was amplified from genomic DNA and cloned into the pGL3‐Basic vector. Both firefly and Renilla luciferase activities were assayed using a dual‐luciferase system (Promega) according to the manufacturer's protocol.

### 2.13 Chromatin immunoprecipitation (ChIP) assay

ChIP was performed using the Magna ChIP™ A/G Chromatin Immunoprecipitation Kit (Merck Millipore Corporation) according to the manufacturer's protocol. The nuclear DNA extracts were amplified using primers that spanned the IL‐18 promoter region and contained the putative binding sites of p65 and Pin1 (Table S1). The re‐ChIP assay was performed to verify whether Pin1 and p65 simultaneously occupied the same binding sites on the IL‐18 promoter. In brief, after the standard ChIP procedure, the chromatin from the beads was eluted by 190 volumes of 10 mmol/L DTT for 30 minutes at 37°C. The eluent was then diluted with sonication buffer before undergoing the ChIP process again.

### 2.14 Statistics

Data are presented as the means ± SD. The data were analysed using SPSS 22.0 software (Abbott Laboratories), and independent Student's tests (two‐tailed) and one‐way ANOVA tests were used to analyse the data between groups. Pearson correlation analysis was used to determine the correlation between the expression level of IL‐18 and RELA/p65 in the TCGA cohorts. Spearman correlation analysis was used to determine the correlation between the PIN1 and IL‐18 expression levels. *P* < .05 was considered significant.

## 3 RESULTS

### 3.1 Pin1 potentially regulates NF‐κB cascade and IL‐18 expression in pancreatic cancer cells

**FIGURE 1:**
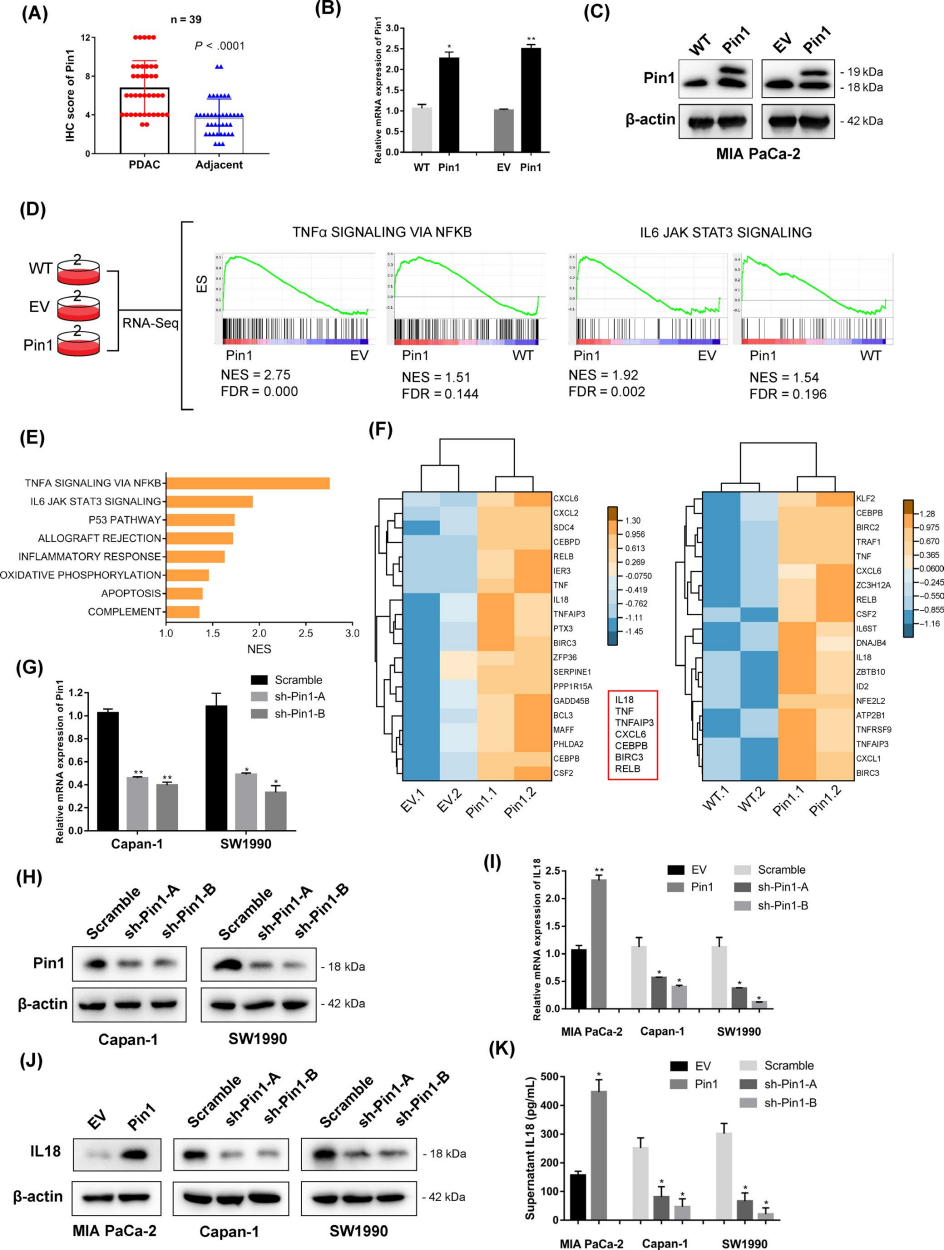
Pin1 potentially regulates NF‐κB cascade and IL‐18 expression in pancreatic cancer cells. (A) Pin1 expression in PDAC and adjacent normal tissues, as determined by the IHC score (n = 39, *P* < .0001). (B) Analysis of relative gene expression data of Pin1 using real‐time quantitative PCR and the 2−ΔΔC_t_ method in MIA PaCa‐2 cell lines. (C) Analysis of protein expression of Pin1 using Western blotting assay in MIA PaCa‐2 cell lines. (D) Transcriptome strategy of RNA sequencing conducted on MIA PaCa‐2 cells. Each group contains two biological replicates. Gene set enrichment analysis (GSEA) was used to analyse the signalling pathways enrichment in different groups. Normalized enrichment score (NES) indicated the analysis results across gene sets. False discovery rate (FDR) presented if a set was significantly enriched. ES, enrichment score. (E) The core‐enriched increased signalling pathways in Pin1‐overexpressing cells compared with EV control. The NES values of the pathways with *P* < .05 are presented. (F) Heat maps show upregulation of “TNFα SIGNALING VIA NFKB” response genes in Pin1‐overexpressing cells. Values are row‐scaled to show relative expression. Blue and yellow are low and high levels, respectively. Red rectangular box showed the mutual upregulated genes in Pin1‐overexpressing cells comparing to WT and EV, respectively. (G) qRT‐PCR analysis of Pin1 knockdown efficiency in Capan‐1 and SW1990 cell lines that stably express shRNA oligos against Pin1. (H) Western blot analysis further confirmed the silencing efficacy. (I) The IL‐18 mRNA levels in PDAC cells were determined following the silencing or overexpression of Pin1 and compared with the controls. (J) The expression of IL‐18 was determined by Western blot analysis. (K) Supernatant was collected and analysed by ELISA for IL‐18 protein expression after cultivation for 24 h. *P < .05, **P < .01

Our previous research showed the oncogenic role of Pin1 in supporting pancreatic cancer growth by maintaining redox balance via c‐Myc/NRF2 axis and highlighted it as an independent prognostic marker for PDAC.^16^ We confirmed that Pin1 was elevated in PDAC tissue comparing to the adjacent normal tissue in 39 patients with PDAC (Figure 1A; Figure S1A). This study focused on the oncogenic role of Pin1 in other malignant biological behaviours in PDAC. Therefore, we stably established stable MIA PaCa‐2 cells that ectopically overexpressed Pin1 based on comparison to expression from pancreatic cancer cell lines detected in our previous research (Figure 1B,C).^16^ RNA sequencing was applied to examine the gene expression profiles of wide‐type (WT) cells, empty vector (EV)–transfected cells and Pin1‐overexpressing MIA PaCa‐2 cells to explore a potential target.^16^ Then, we analysed the different groups based on data sets in GSEA. Of all the hallmarks, “TNFA_SIGNALING_VIA_NFKB” and “IL6_JAK_STAT3_SIGALING” displayed the highest normalized enrichment score (NES) with *P* < .001 in comparison between Pin1‐overexpression and EV/WT groups (Figure 1D). In addition, the highly expressed genes were clearly categorically linked to altered signalling pathways (Figure 1E). We next focused on the top 20 genes displayed core enrichment in “TNFA_SIGNALING_VIA_NFKB” data sets in both compared groups, and the differential gene expression was shown in the clustering heat map. We observed that several mutual genes in the two heat maps, including IL‐18, TNF, TNFAIP3 and so forth, indicating that they may be strongly involved in high Pin1 expression‐associated NF‐κB signalling (Figure 1F). To validate our findings, we additionally stably transfected Capan‐1 and SW1990 cells with Pin1‐specific shRNA based on our previous research concerning the role of Pin1 in PDAC (Figure 1G,H). The result of qPCR showed increased mRNA levels of IL‐18 in Pin1‐overexpressing MIA PaCa‐2 cells and decreased expression in Pin1‐knockdown Capan‐1 and SW1990 cells (Figure 1I). Furthermore, the Pin1‐overexpressing MIA CaPa‐2 cell line displayed upregulated expression of IL‐18 protein, while the Pin1‐knockdown Capan‐1 and SW1990 cells showed significantly decreased IL‐18 expression (Figure 1J), so did the secreted IL‐18 levels in cell culture supernatants (Figure 1K). Thus, we found that Pin1 potentially activated NF‐κB signalling and correlated with IL‐18 expression in pancreatic cancer cells.

### 3.2 Pin1 positively correlates with IL‐18 expression in pancreatic cancer patients

**FIGURE 2:**
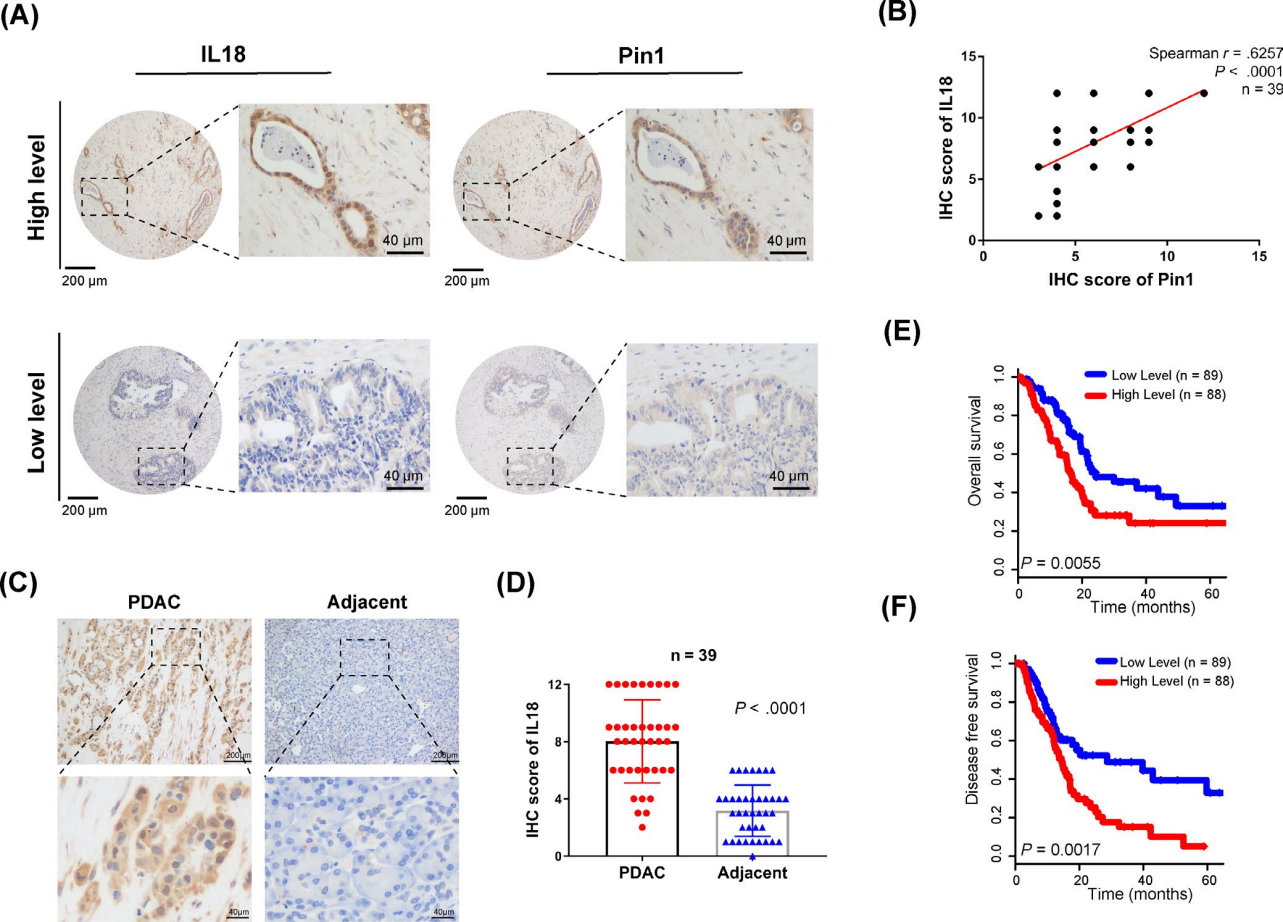
Pin1 positively correlates with IL‐18 expression in pancreatic cancer patients. (A) Representative images of IHC staining for Pin1 and IL‐18 in pancreatic cancer tissues (scale bar, 200 µm; inset scale bar, 40 µm). (B) Correlation analysis of Pin1 expression and IL‐18 expression in PDAC tissues, as determined by the IHC score (n = 39, *P* < .0001). (C) Representative images of IHC staining for IL‐18 in PDAC and adjacent normal tissues (scale bar, 200 μm; inset scale bar, 40 µm). (D) IL‐18 expression in PDAC and adjacent normal tissues, as determined by the IHC score (n = 39, *P* < .0001). (E‐F) The prognostic value of IL‐18 showed that high expression of IL‐18 predicted a worse prognosis for pancreatic cancer by analysis of the TCGA data set

We next examined whether Pin1 expression is correlated with IL‐18 expression in PDAC patients. IHC staining indicated that Pin1 expression was positively correlated with IL‐18 expression in PDAC tissues in 39 patients diagnosed with PDAC in our cancer centre (Figure 2A,B). In addition, IL‐18 were elevated in PDAC tissues compared to adjacent normal tissues (Figure 2C,D). Moreover, we performed a survival analysis based on the TCGA data set and found that IL‐18 was an independent prognostic factor for overall survival (OS) and disease‐free survival (DFS) in the TCGA‐PAAD cohort (Figure 2E,F).

### 3.3 Pin1 promotes and participates in IL‐18‐induced oncogenic behaviour in pancreatic cancer cells

**FIGURE 3:**
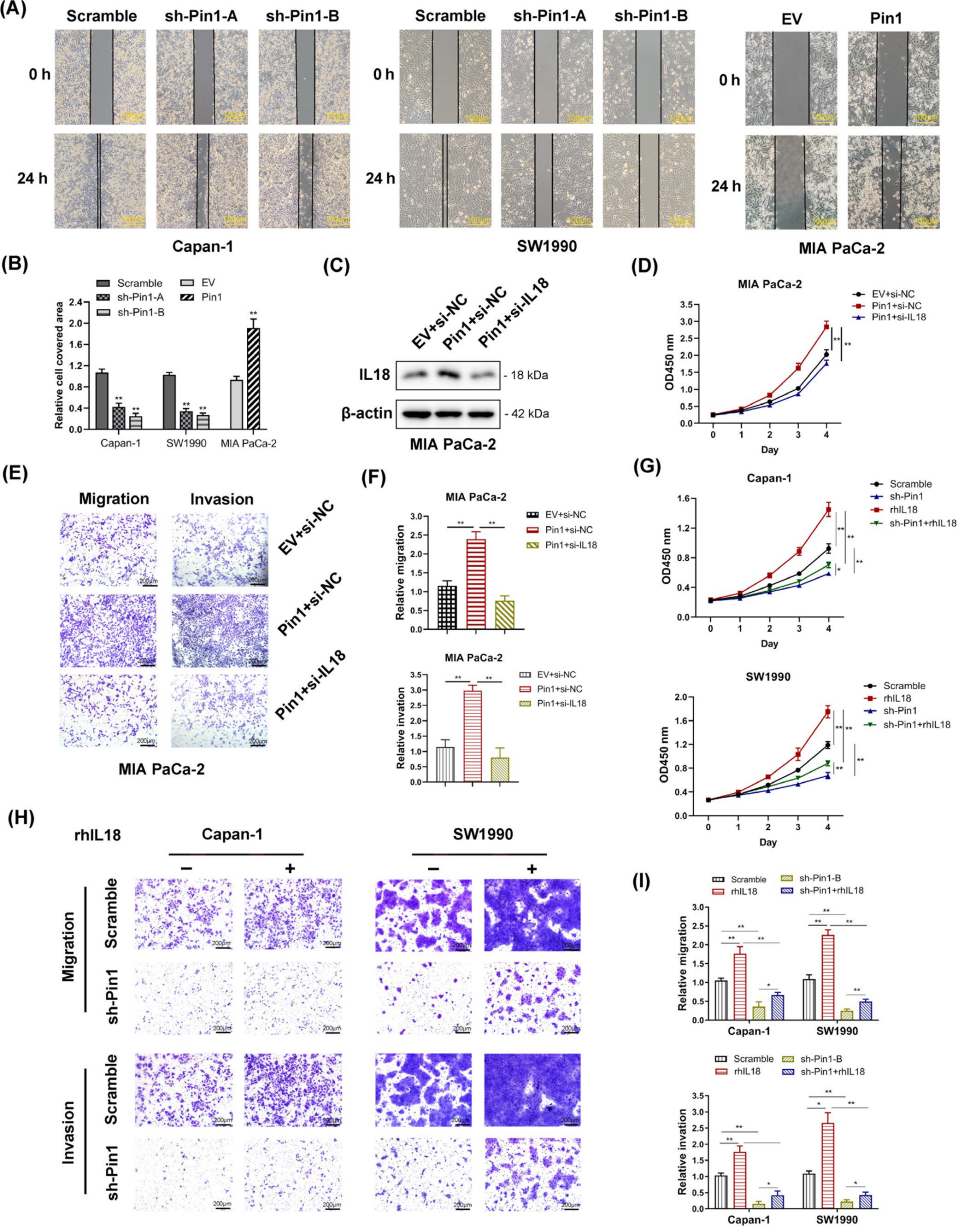
Pin1 promotes and participates in IL‐18‐induced oncogenic behaviour in pancreatic cancer cells. (A) Pin1‐silenced Capan‐1 and SW1990 cells both exhibited significantly decreased cell motility, while Pin1‐overexpressed MIA PaCa‐2 cells dramatically increased cell motility in the wound healing assay (scale bar, 100 μm); quantitation of the data is shown in (B). (C) The expression of IL‐18 was determined by Western blot. (D) A CCK‐8 assay was used to detect the proliferation of MIA PaCa2 cells overexpressing Pin1 and Pin1‐overexpressing cells transfected with IL‐18 siRNA. Knockdown of IL‐18 by siRNA reversed the proliferation ability enhanced by Pin1 overexpression. (E) Transwell assays were applied to measure the migration and invasiveness of MIA PaCa2 cells overexpressing Pin1 and transfected with IL‐18 siRNA. Knockdown of IL‐18 by siRNA decreased both the capacity of migration and invasion induced by Pin1 overexpression; quantitation of the data is shown in (F). (G) Pin1 knockdown cells and scrambled control were treated with rhIL‐18 (20 ng/mL) or PBS for 36 h and subjected to the CCK‐8 assay. (H) Pancreatic cancer cells were treated with rhIL‐18 (20 ng/mL) or PBS for 36 h and subjected to the transwell assay. Knockdown of Pin1 reversed the capacity of migration and invasion induced by IL‐18, while IL‐18 saved the capacity of migration and invasion decreased by Pin1 silencing (scale bar, 200 μm). (I) The relative cell number of migration was quantitated and shown as means ± SD. *P < .05, **P < .01

Pin1 is a peptidyl‐prolyl *cis/trans* isomerase that functions in the oncogenic processes of various tumour cells. We performed wound healing and transwell assays to assess the role of Pin1 in pancreatic cancer cell motility. Our data indicated that Pin1 silencing significantly inhibited the extent of wound closure, while Pin1 overexpressing exhibited the opposite result (Figure 3A,B). Consistently, the results also revealed that Pin1‐silenced Capan‐1 and SW1990 cells exhibited decreased migration and invasion, while Pin1‐overexpressing MIA PaCa‐2 cells showed increased migration and invasion (Figure S2A,B). To investigate the function of IL‐18 in Pin1‐induced cancer progression, we transfected the IL‐18 siRNA into the MIA PaCa‐2 cells stably overexpressed Pin1 (Figure 3C). We observed that IL‐18 silencing dramatically counteracted the enhanced cell proliferation caused by elevated Pin1 expression and found IL‐18 siRNA completely counteracted the cancer‐promoting effect of Pin1 (Figure 3D,E). Similarly, the subsequent transwell assay indicated that silencing IL‐18 reversed the tumour‐promoting effect of Pin1 in cell migration and invasion (Figure 3E,F). In addition, recombination human IL‐18 (rhIL‐18) enhanced the proliferation and invasiveness of pancreatic cancer cells, whereas this tumour‐promoting effect were eliminated by Pin1 knockdown. Moreover, rhIL‐18 also rescued Pin1‐knockdown induced repression of proliferation and metastasis in pancreatic cancer cells (Figure 3G‐I). These results suggested that Pin1 and IL‐18 might form a feedback loop in the oncogenic activity of pancreatic cancer cells.

### 3.4 Pin1 binds to p65 and facilitates NF‐κB activation in pancreatic cancer cells

**FIGURE 4:**
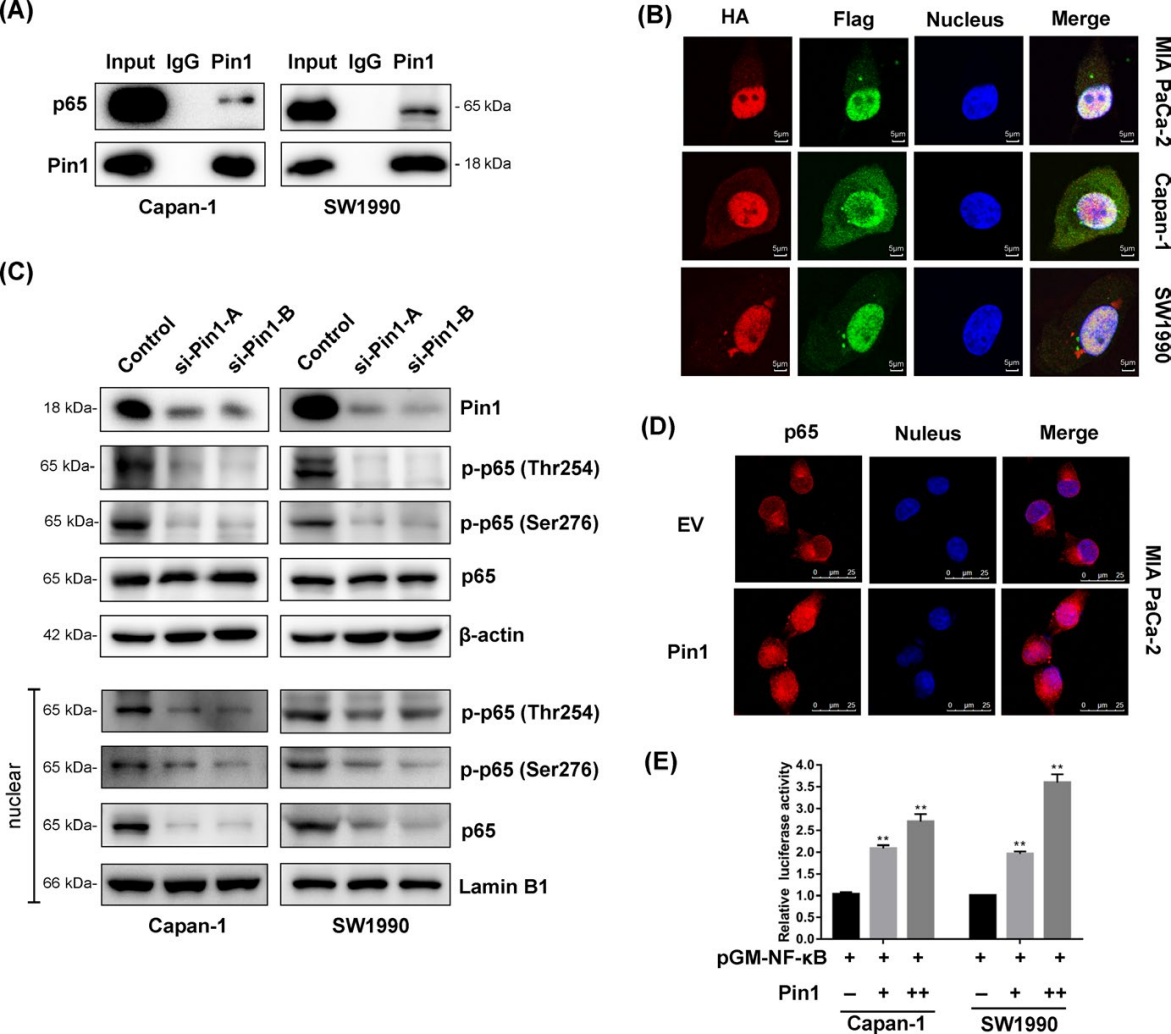
Pin1 binds to p65 and facilitates NF‐κB activation in pancreatic cancer cells. (A) Co‐immunoprecipitation analysis of the interaction between Pin1 and p65 in pancreatic cancer cells. (B) Double immunofluorescent staining revealed co‐localization of the Pin1 and p65 proteins in Capan‐1 and SW19990 cells (scale bar, 5 μm). (C) The effects of Pin1 knockdown on phosphorylation of p65 were assessed by Western blot. (D) Nuclear translocation of p65 induced by Pin1. MIA PaCa‐2 cells were transfected with Pin1 or empty vector, and p65 location was determined (scale bar, 25 μm). (E) Increase of NF‐κB by Pin1 upregulation. Cells were cotransfected with Pin1 vector or control and NF‐κB‐Luc construct, followed by luciferase assay

Pin1 has been identified as a positive regulator of p65 in some cancer cells.^15^, ^26^ To determine whether Pin1‐p65 interaction occurs in pancreatic cancer cells, endogenous CoIP was performed. The results showed that endogenous Pin1 interacted with p65 in Capan‐1 and SW1990 cells (Figure 4A). In addition, immunofluorescence analysis confirmed that cotransfected HA‐Pin1 and Flag‐p65 were predominantly colocalized in MIA PaCa‐2, Capan‐1 and SW1990 cells (Figure 4B). The phosphorylation of p65 has also been altered by differential Pin1 expression. Pin1 silencing significantly decreased the levels of p‐p65 (Thr254) and p‐p65 (Ser276) expression in Capan‐1 and SW1990 cells. We further discovered that the nuclear expression of p65 and p‐p65 was also significantly altered by Pin1 expression, while the overall cellular expression of p65 remained unchanged, which indicated the role of Pin1 in p65 translocation and following transcriptional activity upon phosphorylation (Figure 4C). An immunofluorescence assay also confirmed that overexpression of Pin1 enhanced the translocation of cytoplasmic p65 into the nucleus of MIA PaCa‐2 cells (Figure 4D). We next assessed the impact of Pin1 on NF‐κB transcriptional activity, which was demonstrated by NF‐κB‐luciferase assay. The dual‐luciferase assay showed that NF‐κB activity was increased by Pin1 expression in a dose‐dependent manner in Capan‐1 and SW1990 cells (Figure 4E; Figure S3A).

### 3.5 p65 transcriptionally activates IL‐18 expression in pancreatic cancer cells

**FIGURE 5:**
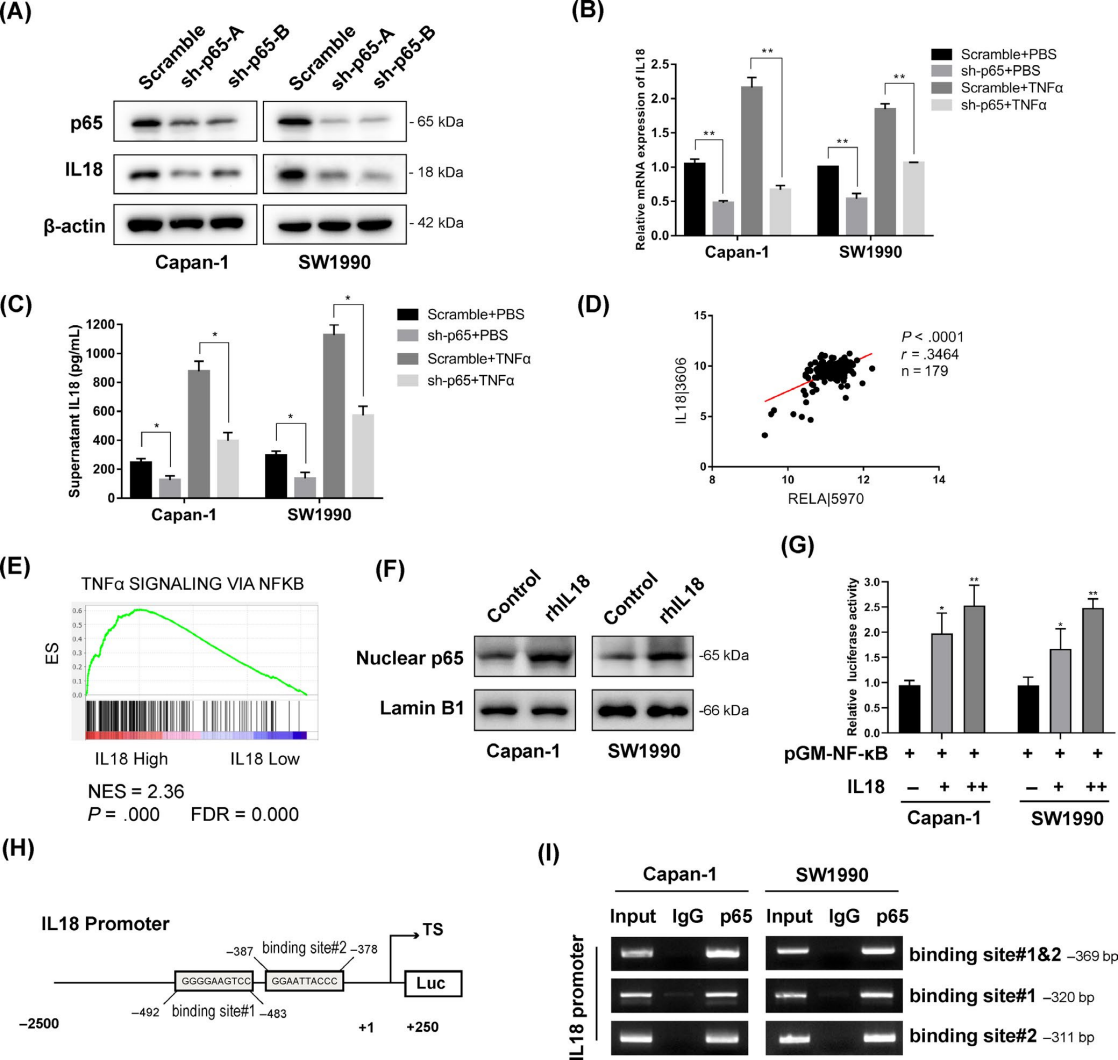
p65 transcriptionally activates IL‐18 expression in pancreatic cancer cells. (A) The expression of p65 and IL‐18 was determined by Western blot analysis. (B) Scramble and sh‐p65 cells were stimulated with TNFα (25 ng/mL) or PBS for 4 h. Total RNA was isolated, and qRT‐PCR was performed to determine the relative IL‐18 mRNA expression (*P < .05, **P < .01). (C) Scramble and sh‐p65 cells were stimulated with TNFα (25 ng/mL) or PBS for 24 h. Supernatant was collected and analysed by ELISA for IL‐18 protein expression (*P < .05). (D) Positive correlation between the expression of RELA/p65 and the expression of the IL‐18 genes in TCGA database. (E) Gene set enrichment analysis (GSEA) was used to analyse the signalling pathways enrichment in different groups based on IL‐18 gene expression level in TCGA database. Normalized enrichment score (NES) indicated the analysis results across gene sets. False discovery rate (FDR) presented if a set was significantly enriched. ES, enrichment score. (F) Western blot analysis of nuclear p65 protein from cells treated with rhIL‐18. (G) Increase of NF‐κB by IL‐18 upregulation. Cells were cotransfected with IL‐18 vector or control and NF‐κB‐Luc construct, followed by luciferase assay. (H) Schematic representation of the IL‐18 promoter regions has shown that IL‐18 promoter region contains putative p65‐binding elements. (I) ChIP assay results with a p65 antibody demonstrated that p65 occupied the promoter region, which contained p65 binding sites

To understand the role of p65 in IL‐18 expression, stable p65 knockdown Capan‐1 and SW1990 cell lines were generated. Stable p65 knockdown in both cell lines resulted in decreased expression of IL‐18 protein (Figure 5A). After treatment with TNFα for 4 hours, the IL‐18 mRNA levels were increased in the presence of p65, but these effects were significantly alleviated in p65 knockdown cell lines (Figure 5B). Furthermore, while TNFα treatment stimulated the expression of secreted IL‐18 protein in the presence of p65, the secreted protein levels were diminished in the p65 knockdown cell lines (Figure 5C). Finally, we validated the correlation between p65 and IL‐18 with data from the TCGA‐PAAD cohort. The correlation analysis showed that RELA/p65 was significantly positively correlated with IL‐18 in the TCGA‐PAAD cohort (Figure 5D). In addition, the GSEA analysis found that the high IL‐18 expression‐associated signatures “TNFA_SIGNALING_VIA_NFKB” exhibited the highest NES, with *P* < .001 (Figure 5E), which indicated that high IL‐18 is strongly correlated to elevated NF‐κB pathway. The following Western blot analysis indicated that stimulating Capan‐1 and SW1990 cells with rhIL‐18 increased nuclear p65 expression (Figure 5F). The dual‐luciferase assay indicated that NF‐κB activity was increased by IL‐18 expression in a dose‐dependent manner in Capan‐1 and SW1990 cells (Figure 5G; Figure S3B). These results suggest that p65 may be critical in Pin1‐IL‐18 feedback loop. Notably, in the IL‐18 promoter, there exists a conserved p65 binding element that is adjacent to an element that is not relatively conserved (Figure 5H). Therefore, we designed primers covered both binding sites and conducted ChIP assays to explore whether p65 could occupy one of these binding sites. ChIP results showed that p65 could interact with the region, and further ChIP assay demonstrated that p65 could occupy either of the two binding elements (Figure 5I).

### 3.6 Pin1 cooperates with p65 in regulation of IL‐18 expression in pancreatic cancer cells

**FIGURE 6:**
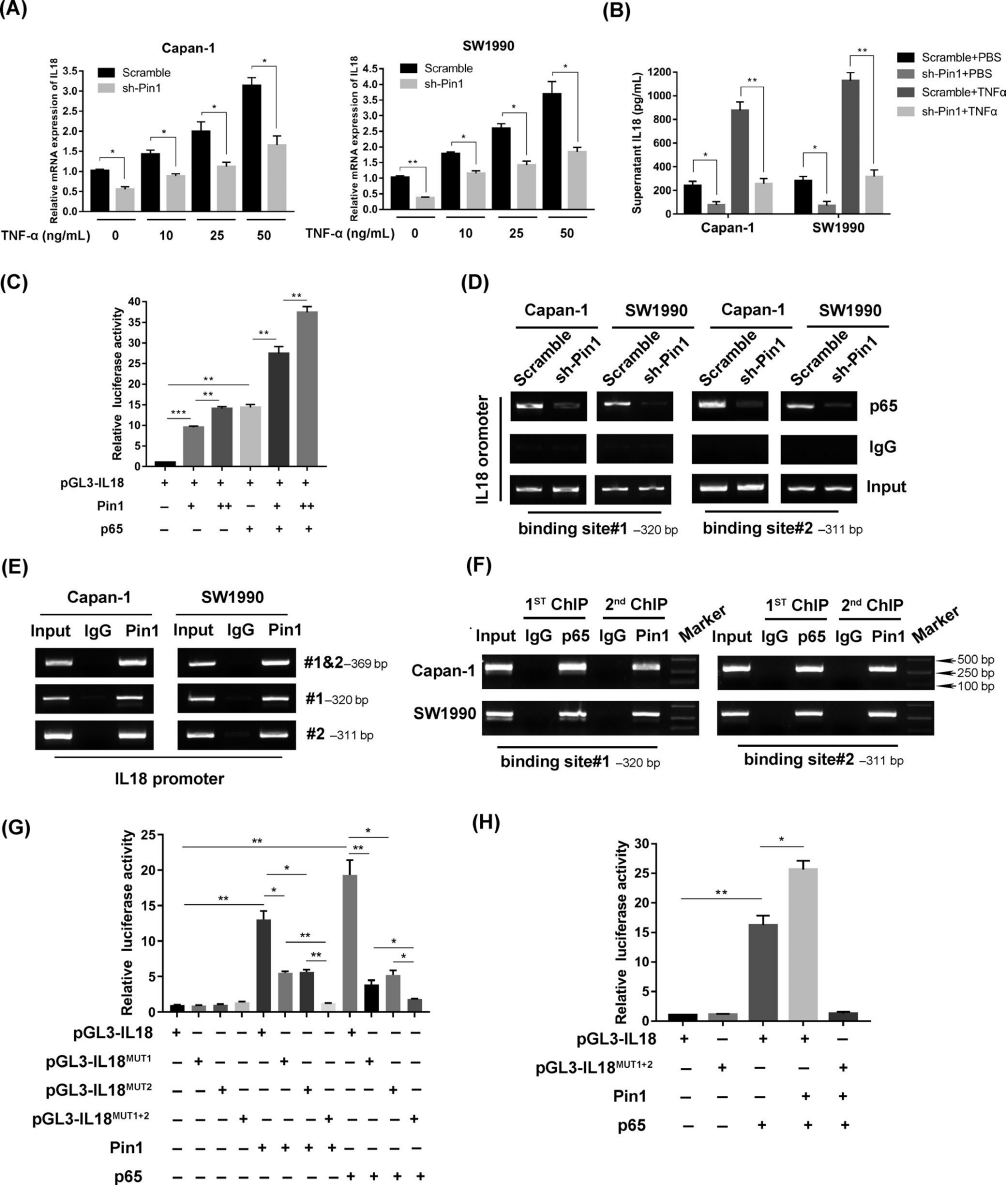
Pin1 cooperates with p65 in regulation of IL‐18 expression in pancreatic cancer cells. (A) Scramble and sh‐Pin1 cells were stimulated with TNFα or PBS for 4 h. Total RNA was isolated, and qRT‐PCR was performed to determine the relative IL‐18 mRNA expression (*P < .05). (B) Scramble and sh‐Pin1 cells were stimulated with TNFα (25 ng/mL) or PBS for 24 h. Supernatant was collected and analysed by ELISA for IL‐18 protein expression (*P < .05, **P < .01). (C) Pin1 increased IL‐18 luciferase activity and p65‐related IL‐18 luciferase activity in a dose‐dependent manner in HEK‐293T cells (**P < .01, ***P < .001). (D) Downregulation of Pin1 decreased the abundance of p65 that occupied the IL‐18 promoter. (E) Pin1 enrichment at the IL‐18 promoter was measured using a Pin1 antibody to perform the ChIP assay. (F) The ChIP and re‐ChIP experiments indicated that Pin1 and p65 synergistically occupied the same promoter region on the IL‐18 promoter. (G) Mutation of IL‐18 promoter region based on the two binding sites decreased the positive effect of Pin1 and p65 on IL‐18 luciferase activity (**P < .01, ***P < .001). (H) Mutation of both binding sites on IL‐18 promoter diminished the positive effect of Pin1 on p65‐related IL‐18 luciferase activity (**P < .01, ***P < .001)

To confirm that Pin1 influences IL‐18 expression through NF‐κB signalling, we performed qPCR to analyse the mRNA expression of TNFα‐induced IL‐18. Our results indicated that the mRNA levels of IL‐18 were increased by TNFα in a dose‐dependent manner, while IL‐18 mRNA levels were inhibited by Pin1 knockdown following treatment with all doses of TNFα in Capan‐1 and SW1990 cell lines (Figure 6A). ELISA results showed that the protein expression of IL‐18 was also more dramatically decreased in Pin1 knockdown cell lines than it was in the scramble‐treated cell lines following TNFα treatment (Figure 6B). We cloned the IL‐18 promoter region into a luciferase vector and then cotransfected it with a Pin1 expression vector and/or a p65 expression vector. The dual‐luciferase assay showed that Pin1 enhanced the IL‐18 promoter activity in a dose‐dependent manner. Moreover, comparing to p65 vector alone, the luciferase activity of IL‐18 promoter increased when cotransfecting Pin1 with p65. The result suggested that Pin 1 might potentially activate the transcriptional activity of p65 on IL‐18 promoter (Figure 6C; Figure S4A). To find the interaction between Pin1 and p65 with the chromatin at the promoter region for IL‐18, we found a decreased occupation of p65 on the IL‐18 promoter in the Pin1 knockdown group (Figure 6D), which suggested that Pin1 influenced the IL‐18 transcription through p65 activity. Pin1 and p65 were predominantly colocalized in Capan‐1 and SW1990 cell lines; thus, we deduced that Pin1 and p65 might interact at the IL‐18 promoter. ChIP results showed that Pin1 could also occupy either of the two binding elements where p65 were able to bind (Figure 6E). The following re‐ChIP assay showed that Pin1 and p65 occupied the same region of the IL‐18 promoter (Figure 6F). Subsequent luciferase assays demonstrated that when either one of the sequences of the two binding sides was mutated, the positive impact of Pin1 and p65 on the mutated luciferase reporter decreased. The positive effect was completely lost when both sequences were mutated (Figure 6G,H; Figure S4B,C).

**FIGURE 7:**
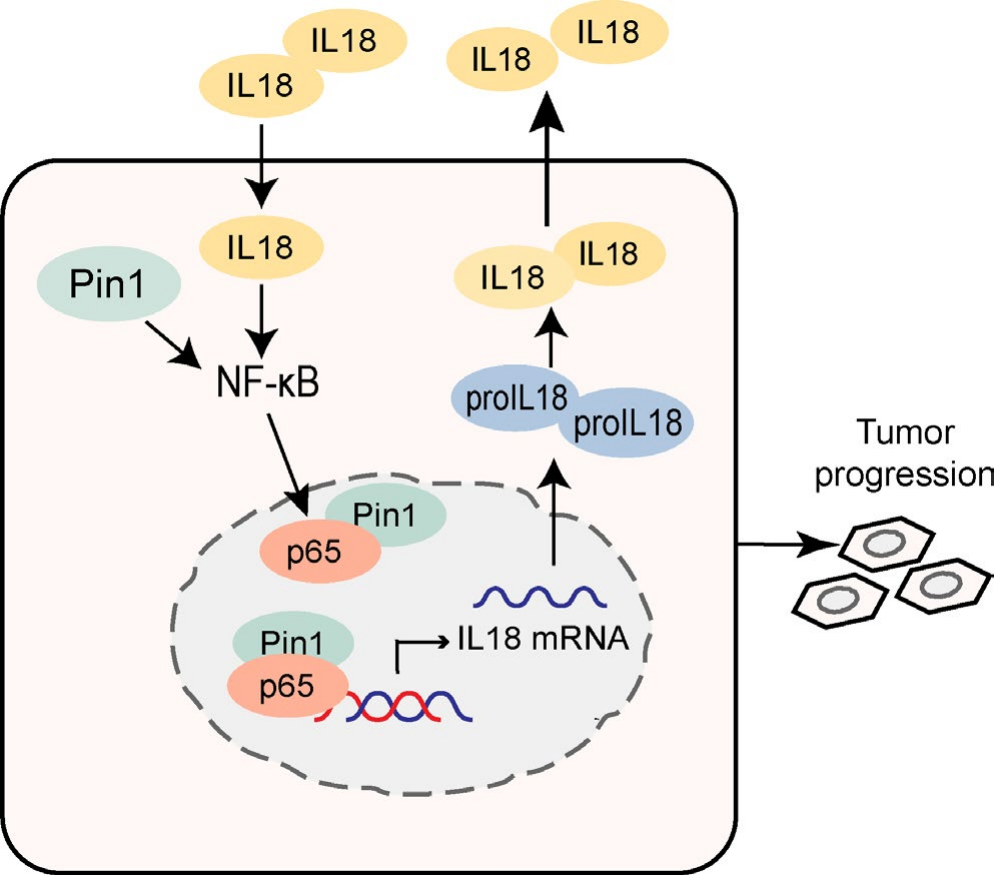
Schematic illustration summarizing the Pin1‐NFκB‐IL‐18 feedback loop in pancreatic cancer cells

In summary, our present study highlights the role of Pin1 in the oncogenic behaviour of PDAC. Mechanist studies demonstrated that Pin1 promotes IL‐18 activation through NF‐κB signalling. The interaction between Pin1 and p65 results in p65 nuclear translocation, leading to increased IL‐18 transcription, expression and secretion, which in turn enhances NF‐κB activation; thus, the feedback loop is formed (Figure 7).

## 4 DISCUSSION

In this study, we elucidated the role of Pin1 on IL‐18 expression via the NF‐κB signalling pathway in PDAC. Consistent with our previous research and with the results from Chen's study,^16^, ^17^ we found that the expression of Pin1 is elevated in PDAC compared to adjacent normal tissues. We previously demonstrated that elevated Pin1 expression in PDAC was an indicator of a poor prognosis and a sign of a maintained redox balance via the c‐Myc/NRF2/ARE axis, which provides antioxidation protection in Kras‐mutant pancreatic cancer cells.^16^ Other Pin1‐mediated mechanisms have been discovered in other tumour types. Pin1 promotes cell proliferation by upregulating cyclins in hepatocellular carcinoma cells.^27^ Pin1 also interacts with Smad3 to drive oncogenic TGFβ signalling and promote breast cancer progression.^28^ Our RNA sequencing results from the different Pin1‐expressing cell lines provide support for the idea that Pin1 might implement its oncogenic effect by regulating NF‐κB signalling in PDAC.

Over fifty molecules have been identified as downstream targets for Pin1, including NF‐κB p65.^12^ Overexpression of Pin1 in cancer cells and tissues has been proved to be correlated with enhanced NF‐κB activation and malignant biological properties in human hepatocellular carcinoma, glioblastoma and breast cancer.^15^, ^29^, ^30^ In human hepatocellular carcinoma cells, Pin1 binds to p‐NF‐κB‐p65 (Thr254) and stimulates its phosphorylation at Ser276, which is followed by translocation of activated NF‐κB into the nucleus. The current study confirmed that Pin1 bound to the NF‐κB p65 protein in PDAC and mediated its phosphorylation (Thr254 and Ser276), which promoted the nuclear translocation of NF‐κB and led to the activation of NF‐κB signalling.

One of our pivotal findings is that Pin1 regulates the gene expression of the chemokine IL‐18. The tumour microenvironment is critical for immune cell regulation and the production of inflammatory cytokines and chemokines. It functions by influencing, integrating and transmitting information regarding inflammatory and immune processes.^31^, ^32^ NF‐κB has been widely recognized as a key bridge linking inflammation with tumorigenesis. The activation of NF‐κB leads to cytokine production, especially IL‐6, IL‐11 and IL‐22, providing an inflammatory environment for the growth of premalignant tumours. Additionally, IL‐6 and IL‐11 activate STAT3 signalling, which works with NF‐κB to promote cancer development.^4^, ^33^, ^34^ IL‐18, a pro‐inflammatory cytokine, was found to be downregulated by PPAR‐γ via inhibition of NF‐κB activity, thus influencing the expression of IL‐18‐induced adhesion molecules.^35^ The simultaneously increase in NF‐κB and IL‐18 levels due to interferon‐γ in pancreatitis also provides a possible relationship between the factors and pancreatic diseases.^36^ Pin1 is an enzyme with two domains. N‐terminal amino acids 1‐19 comprise the WW domain, which determines substrate specificity, while the C‐terminus contains the catalytic prolyl isomerase (PPIase) domain.^37^, ^38^ As a member of the PPIase family, Pin1 binds to and *cis/trans* isomerizes a specific subset of Ser/Thr‐Pro in its target proteins. Phosphorylation‐dependent isomerization by Pin1 participates in many cellular processes, such as protein interaction, dephosphorylation, subcellular localization and transcription.^11^, ^37^, ^38^ Pin1 has been reported to directly enhance the DNA binding functions of oestrogen receptor α (ERα). Pin1 selectively enhances the binding affinity of ER to consensus DNA elements that Pin1 isomerization of phosphorylated ERα directly regulates the function of the adjacent DNA binding domain, and this interaction is further modulated by ligand binding in the ligand‐binding domain.^39^ Our study identified two binding sites of Pin1 and p65 in the IL‐18 promoter and confirmed that the two molecules simultaneously bind to the promoter region and enhance transcription. Furthermore, the transcriptional activity of IL‐18 promoter increased upon cotransfection of p65 and Pin1 comparing to p65 alone. Based on the potential isomerization function of Pin1, we supposed that Pin1 might isomerize phosphorylated p65 and regulate the function of its DNA binding domain and following transcriptional activity. Further protein interaction motif screen for discovering the motif that mediated interaction between Pin1 and p65 is required. Moreover, DNA binding analysis is required to analyse the possible Pin1‐dependent allosteric regulation of p65 function. Though IL‐18 could enhance tumour immunity to some extent,^40^ our research found that IL‐18 protein was elevated in tumour tissues and that IL‐18 mRNA expression was a predictor of poor prognoses. IL‐18 was reported to perform an oncogenic role in supporting cancer cell survival and invasion. IL‐18 levels were increased by rhVEGF‐165 via the generation of ROI and ERK1/2 phosphorylation, which promoted migration of gastric cancer cells.^41^ IL‐18 was reported to enhance the metastatic ability of melanoma cells via the generation of ROI and activation of the MAPK pathway.^42^ In agreement with our findings, other data proved that IL‐18 regulates NF‐κB signalling to implement its tumorigenic capacity,^20^ which supports the Pin1/NF‐κB/IL‐18 feedback loop in pancreatic cancer cells.

The most challenging features of PDAC are its early invasion and migration; thus, finding a feasible therapeutic strategy targeting tumour metastasis could assist in improving the prognosis of patients with PDAC.^43^ Pin1 has been reported to promote pancreatic cancer metastasis in vitro and *in vivo*.^17^ In addition, IL‐18 was reported to be capable of stimulating the invasion and metastasis of PDAC and hepatic carcinoma.^20^, ^44^ Our finding was that Pin1 expression was positively correlated with IL‐18 expression. Pin1 promoted cell metastasis by increasing IL‐18 expression, and the oncogenic effect of IL‐18 was also significantly decreased in Pin1 knockdown cells, which highlighted the role of Pin1 in IL‐18‐induced tumour‐promoting effect. IL‐18 can regulate both innate and adaptive immune responses, which have been reported to promote the cytolytic activity of cytotoxic T cells and nature killer (NK) cells^18^, ^20^; further, the safety and activity of rhIL‐18 have been investigated in clinical tries,^45^, ^46^ so we proposed a hypothesis that IL‐18 is a potential therapeutic target when combined with Pin1 inhibition in PDAC. Further studies are needed to validate this speculation.

This study has focused on the underlying mechanisms accounting for the constitutively activated NF‐κB signalling in PDAC. However, the important role of Pin1 in oncogenesis may not be limited to NF‐κB signalling in PDAC. Additionally, we overserved that rhIL‐18 only partially restored reduced malignant behaviours induced by Pin1 knockdown, while Pin1 knockdown could abrogate the rhIL‐18‐induced malignant behaviours. Thus, there might be other factors involved in Pin1 pathway. Pin1 has been reported to enhance the IL‐6/STAT3 pathway in different cancer types.^47^, ^48^ Our RNA sequencing data also showed that Pin1‐upregulated genes were enriched in gene set of “IL6_JAK_STAT3_SIGNALING,” which indicated that Pin1 might be involved in the regulation of STAT3 signalling in PDAC, which requires further investigation.

In summary, our present study elucidated a novel regulatory mechanism for Pin1 via activation of NF‐κB‐mediated inflammation and the importance of Pin1 in oncogenic behaviour in pancreatic cancer cells. Pin1 promoted cancer progression by increasing IL‐18 expression, while Pin1 knockdown impaired IL‐18‐associated proliferation and metastasis. Thus, Pin1 may be critical in balancing the cancer‐promoting and cancer‐suppressing effects of IL‐18. Overall, the Pin1 pro‐inflammatory programme represents an unappreciated mediation of oncogenesis, and development of an inhibitor of the programme would be helpful in prolonging the survival of patients with PDAC.

## CONFLICT OF INTEREST

The authors declare that they have no conflict of interest.

## AUTHORS' CONTRIBUTIONS

YQ, XWX and XJY designed the experiments; QQS, GXF, QFZ and QSH conducted the experiments; SRJ, WYX and WSL provided research materials and methods; QQS, WXD and ZY analysed data; QQS, ZZ and MQL made the figures; QQS wrote the manuscript; YQ and XWX revised the manuscript. All authors read and approved the final manuscript.

## Supporting information

Fig S1

Fig S2

Fig S3

Fig S4

Table S1

## Data Availability

The data that support the findings of this study are available from the corresponding author upon reasonable request.
