# Supplementary figures and images for: Pin1 promotes pancreatic cancer progression and metastasis by activation of NF‐κB‐IL‐18 feedback loop
Source: Cell Prolif. 2020 Apr 29;53(5):e12816. doi: 10.1111/cpr.12816 (PMC7260075; doi:10.1111/cpr.12816)

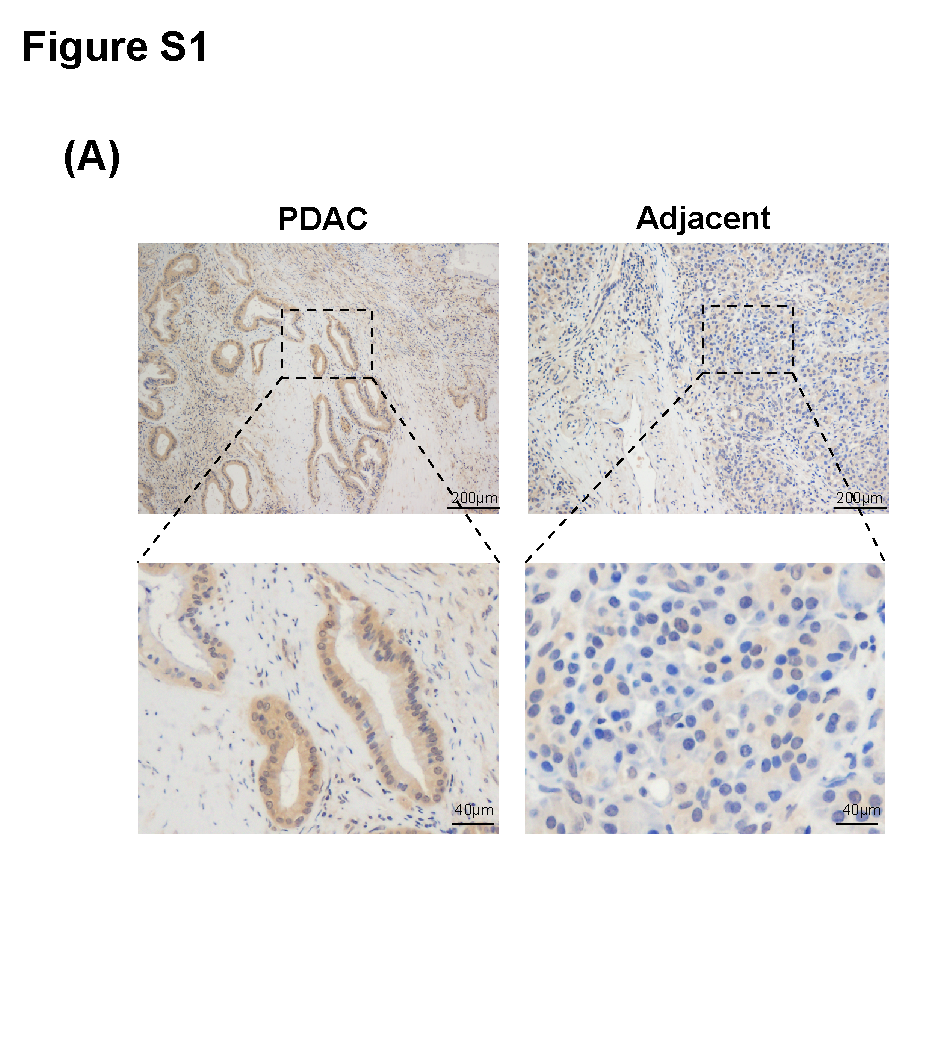

Supplement: Supplementary file 1 — Fig S1 [file CPR-53-e12816-s001.tif]

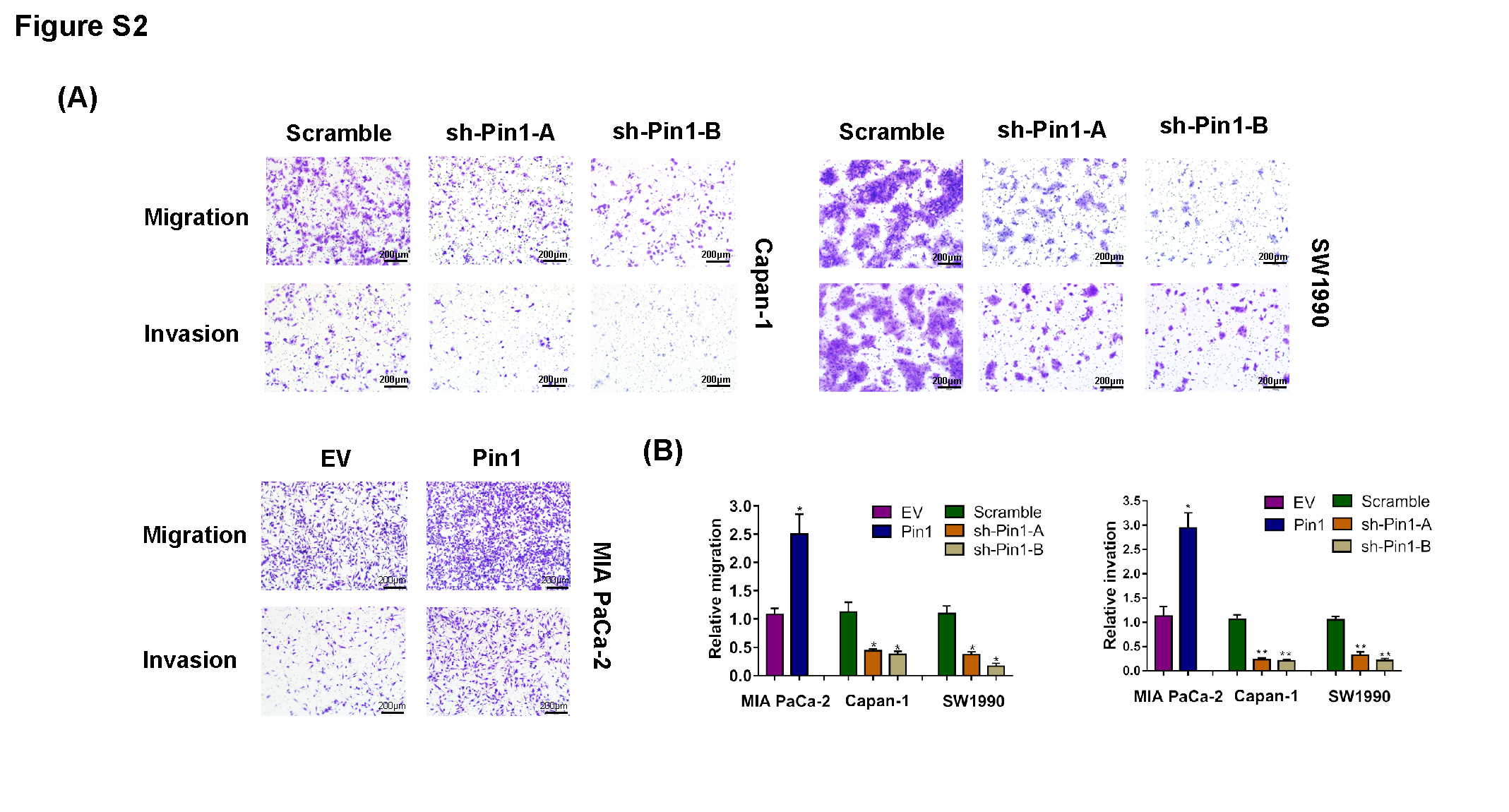

Supplement: Supplementary file 2 — Fig S2 [file CPR-53-e12816-s002.tif]

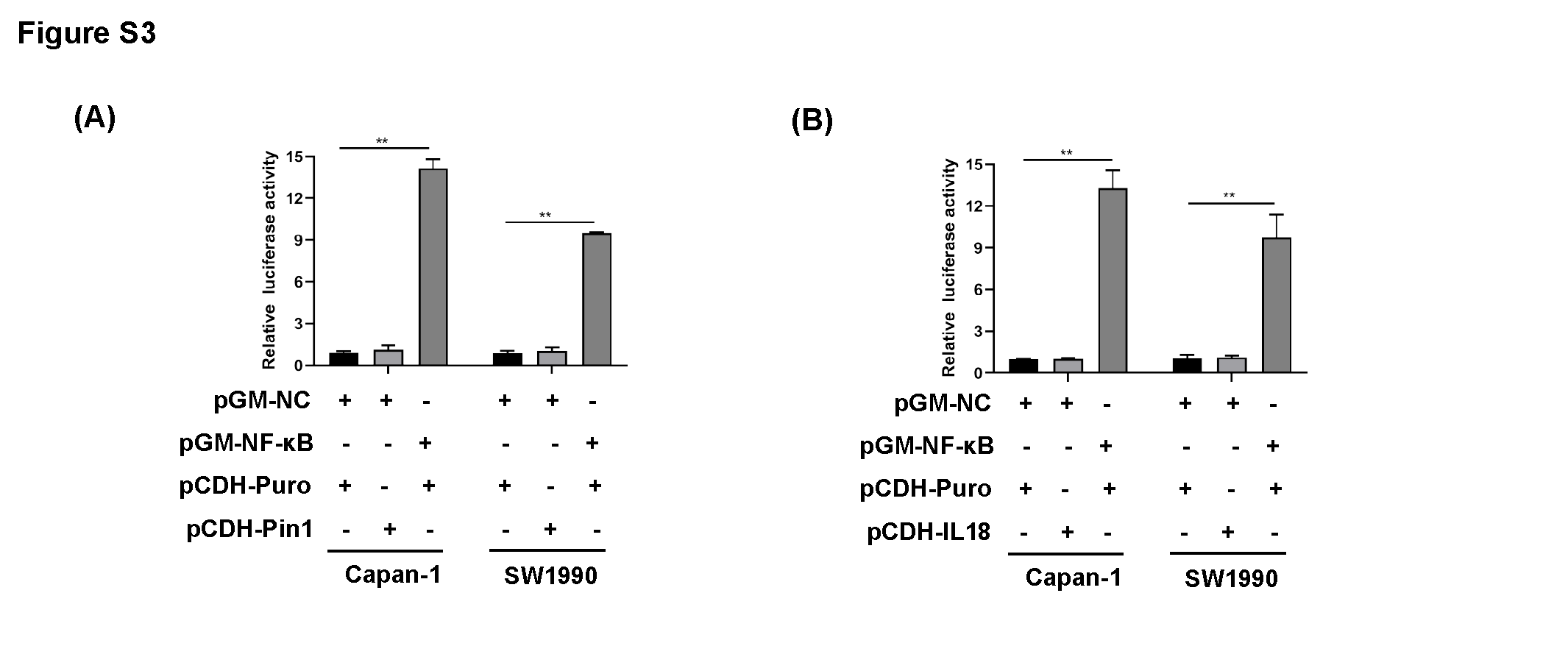

Supplement: Supplementary file 3 — Fig S3 [file CPR-53-e12816-s003.tif]

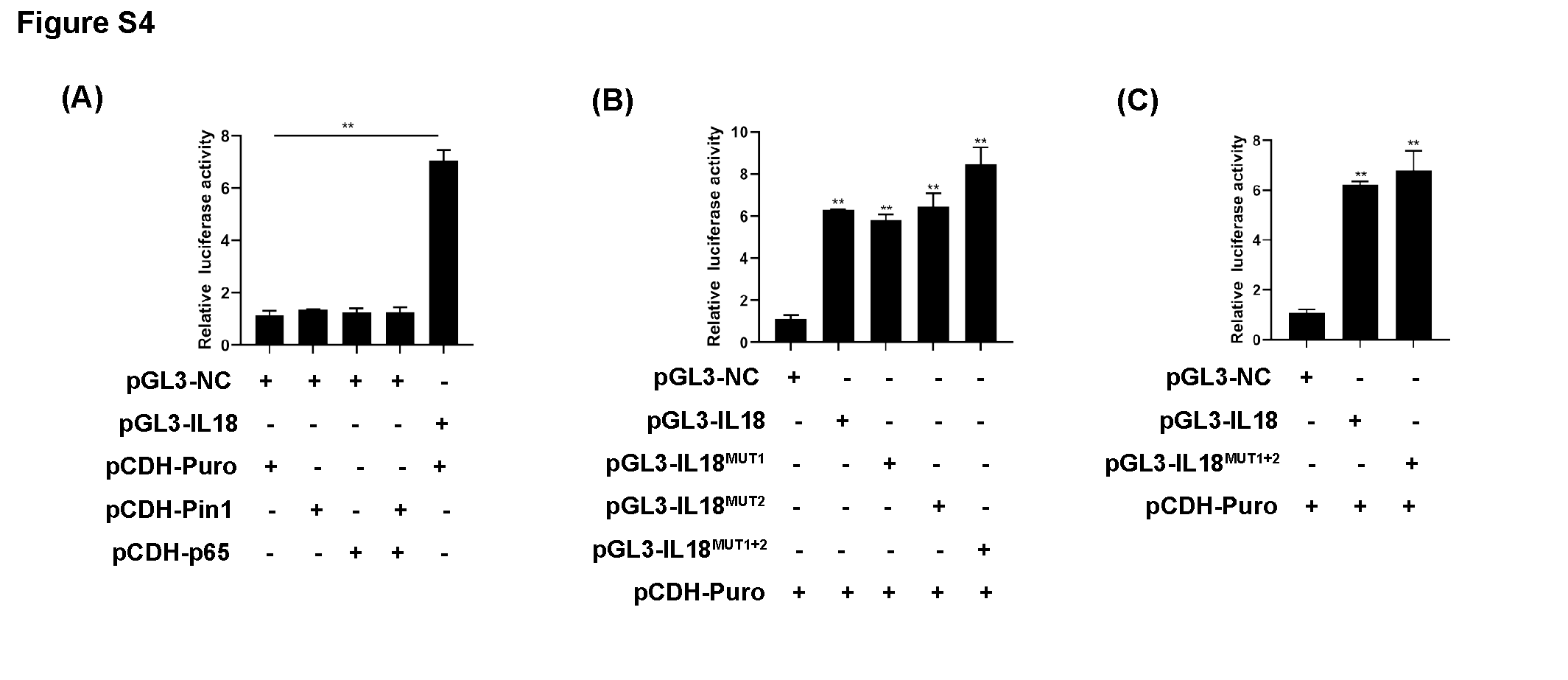

Supplement: Supplementary file 4 — Fig S4 [file CPR-53-e12816-s004.tif]
