# Supplementary material for: Pin1 promotes pancreatic cancer progression and metastasis by activation of NF‐κB‐IL‐18 feedback loop
Source: Cell Prolif. 2020 Apr 29;53(5):e12816. doi: 10.1111/cpr.12816 (PMC7260075; doi:10.1111/cpr.12816)
Supplement: Supplementary file 5 — Table S1 [file CPR-53-e12816-s005.docx]

**Table S1 Primer sequences used in this study**

| *Pin1* forward | 5'- CCTGGAGCTGATCAACGGCTACAT-3' |
| --- | --- |
| *Pin1* reverse | 5'- GGATGATGTGGATGCCGGAATCCG-3' |
| *IL18* forward | 5'- GACCAAGGAAATCGGCCTCTATT-3' |
| *IL18* reverse | 5'- GTATCCTTGATGTTATCAGGAGG-3' |
| *β-actin* forward | 5'- AGAGCTACGAGCTGCCTGAC -3' |
| *β-actin* reverse | 5'- AGCACTGTGTTGGCGTACAG -3' |
| ChIP forward 1 | 5'- TCCGTGTGGGCTTGCATCTGATAC -3' |
| ChIP reverse 1 | 5'- GCACCTCCTTAGTCCATTAGAAGC -3' |
| ChIP forward 2 | 5'- GATGGTATCCGTGTGGCTTGCATC -3' |
| ChIP reverse 2 | 5'- CAGACTTCCTGGTCACACTTCAGC -3' |
| ChIP forward 3 | 5'- TTGAGCTTGGGAGGAAGGGGAAGT -3' |
| ChIP reverse 3 | 5'- GTGCCTTCCAAAACCTGTACCTC -3' |
